# S-ResNet: An improved ResNet neural model capable of the identification of small insects

**DOI:** 10.3389/fpls.2022.1066115

**Published:** 2022-12-22

**Authors:** Pei Wang, Fan Luo, Lihong Wang, Chengsong Li, Qi Niu, Hui Li

**Affiliations:** ^1^ Key Laboratory of Agricultural Equipment for Hilly and Mountain Areas, College of Engineering and Technology, Southwest University, Chongqing, China; ^2^ Key Laboratory of Intelligent Equipment and Robotics for Agriculture of Zhejiang Province, College of Biosystems Engineering and Food Science, Zhejiang University, Hangzhou, China; ^3^ Key Laboratory of Modern Agricultural Equipment and Technology (Jiangsu University), Ministry of Education, School of Agricultural Engineering, Jiangsu University, Zhenjiang, China; ^4^ Interdisciplinary Research Center for Agriculture Green Development in Yangtze River Basin, Southwest University, Chongqing, China; ^5^ National Citrus Engineering Research Center, Chinese Academy of Agricultural Sciences & Southwest University, Chongqing, China

**Keywords:** deep learning in agriculture, small target insect, insect identification, ResNet model, convolutional neural network

## Abstract

**Introduction:**

Precise identification of crop insects is a crucial aspect of intelligent plant protection. Recently, with the development of deep learning methods, the efficiency of insect recognition has been significantly improved. However, the recognition rate of existing models for small insect targets is still insufficient for insect early warning or precise variable pesticide application. Small insects occupy less pixel information on the image, making it more difficult for the model to extract feature information.

**Methods:**

To improve the identification accuracy of small insect targets, in this paper, we proposed S-ResNet, a model improved from the ResNet, by varying its convolution kernel. The branch of the residual structure was added and the Feature Multiplexing Module (FMM) was illustrated. Therefore, the feature expression capacity of the model was improved using feature information of different scales. Meanwhile, the Adjacent Elimination Module (AEM) was furtherly employed to eliminate the useless information in the extracted features of the model.

**Results:**

The training and validation results showed that the improved residual structure improved the feature extraction ability of small insect targets compared to the original model. With compare of 18, 30, or 50 layers, the S-ResNet enhanced the identification accuracy of small insect targets by 7% than that on the ResNet model with same layer depth.

## 1 Introduction

Along with diseases and weeds, insect infestation is an important factor affecting the crop production. The insects could damage crops at all growth stages, which would seriously reduce the yield and quality of crop products (Bao et al., 2021). Therefore, accurate identification of insects and more efficient pest control are essential to increase the growers’ economic interest. The conventional insect identification relies on the visual diagnosis experience of plant protection experts and growers. However, this diagnostic method is time-consuming, inefficient, and highly subjective. The diagnose ability could be insufficient in large scale crop cultivation. Thus, intelligent insect identification system is demanded to apply timely pest control strategies.

Significant progress has been made in insect identification using machine vision technology. For example, Larios et al. (2008) classified stonefly larvae based on Scale-Invariant Feature Transform (SIFT) and the histograms of the target features. Samanta and Ghosh (2012) implemented the diagnosis of eight insect species in tea plantations using correlation-based feature selection and artificial neural networks based on a dataset containing 609 samples. Using Support Vector Machine (SVM), Ebrahimi et al. (2017) identified the thrips in crop canopies. Although the above methods have shown exemplary performance in pest identification, conventional machine learning algorithms must be applied after the procedure of image pre-processing, which was time-consuming for massive data processing in practice. Thus, there were few capable models for pest early-warning or precise control applications.

In recent years, deep learning had received increasing attention from researchers due to its superior performance in feature extraction, model generalization and fitting (Liu et al., 2022). Among them, Convolutional Neural Networks(CNNs) had been used extensively in large-scale image recognition tasks and had achieved good results. Due to the excellent performance of CNNs, they had also been used in pest identification. For example, an improved AlexNet model successfully identified ten insect species in complex farming contexts with an accuracy as high as 98.67% (Cheng et al., 2017). By analyzing the effects of convolutional kernels and layers, Wang et al. (2017) reconstructed a model including the elements from AlexNet and LeNet-5 for the classification of 82 insect species, with an accuracy of 92%. Thenmozhi and Reddy (2019) proposed an effective deep CNNs model to classify insects in three open access datasets. Data augmentation methods such as reflection, scaling, rotation, and panning were introduced to prevent model overfitting. The final classification accuracy of the model achieved 96.75%, 97.47%, and 95.97%, respectively.

With the addition of an pre-trained MobileNet-V2 as a backbone and an attention mechanism, Chen et al. (2021) classified the pests in an open access dataset with the average accuracy of 99.14%. Jiao et al. (2022) introduced a feature enhancement module and an adaptive enhancement module to the R-CNN model. The improved model was tested using the AgriPest21 dataset, achieved a recognition accuracy of 77%. The good feature extraction capability of CNNs allows the model to learn higher-level semantic information. It also benefits the improvement of the model recognition accuracy and robustness, as well as the reduction of human efforts (e.g., manual extraction of features). However, all the above studies used image training resources with high proportion of insect targets in all image pixels, which containing various information of the insect features. In practical photographing procedure during precise insect control process, the insect targets could just occupy a quite low proportion of pixels in the collected images. During the forward propagation of CNNs, the pest information in the learning layer gradually decreases. Therefore, it could be more difficult to extract the appropriate insect features for correct identification.

**Figure 1:**
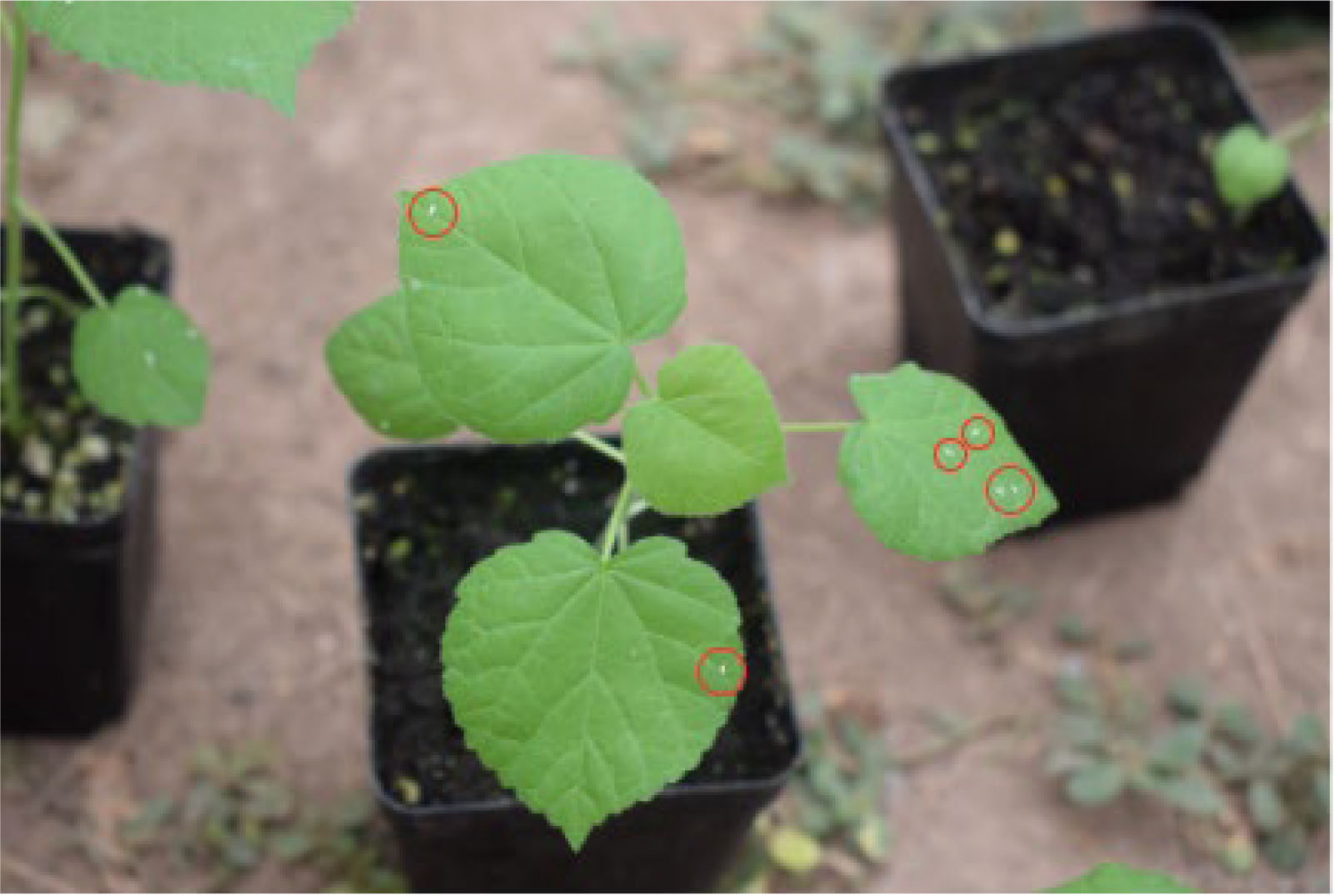
Small inset targets in the actual environment.

In summary, CNNs had been used extensively in the field of insect identification. As their ability to automatically acquire target features from training datasets not only avoided labor-intensive feature engineering and complex image processing processes, but also allowed the model to learn more high-level semantic information which could improve the recognition accuracy and robustness of the model. However, these methods still had some limitations in identifying small target insects. The small target of insects in the real environment (Targets occupied less pixel information on the RGB image, **Figure 1**) led to the problem that CNNs might not be able to extract sufficiently detailed recognition features for the small target insects in the image. It might cause the trained classifiers to be less accurate for insect recognition. Currently, a large number of researchers had also started to focus on the difficult problem of small target insect identification. For example, Wang et al. (2021) proposed Sampling-balanced Region Proposal Network(S-RPN) model. This model firstly added attention module to the residual structure to obtain richer detailed features of small target insects. Secondly, S-RPN reconstructed the Region Proposal Network (RPN) to obtain higher quality target proposals. Finally, S-RPN achieved detection of small target insects and obtained good results. However, the two-stage detection mode took a lot of time to generate proposals, which was not conducive to the real-time detection of small target insects (Wen et al., 2022). Meanwhile its only enriched the insect feature information by the attention mechanism, but the actual pest size was much smaller than the general small target. It might cause the features to fade away with the convolution operation. Therefore, its backbone model was not sufficient for extracting pest feature information. Wen et al. (2022) proposed Pest-YOLO v4 to obtain better detection performance for small target insects by optimizing the loss function and the choice of prediction box. To effectively identify maize insects, Zhang et al. (2022) improved YOLO v4 by designing a multiscale hybrid attention mechanism to improve the model’s focus on small target pests and fuse the effective information of multi-scale additional features. However, all the above methods achieve small target insect recognition by optimizing proposals, loss functions, and fusing contextual features, and rarely enhance the feature capability of small target pests by optimizing the backbone network, which led to the loss of target feature information during the forward propagation of the model. Even though fusing contextual features could effectively utilize multi-level feature information. When features were mixed with small, medium and large targets (background information). The dominance of large and medium targets would weaken the features of the small targets thus leading to the missed identification of the small targets.

An S-ResNet model based on improving ResNet was proposed to solve the difficulty of small target insect feature extraction and the large amount of redundant information mixed in the extracted features in this study. Firstly, the extraction ability of model for small target insects was enhanced by optimizing the convolution kernel and increasing the branches of the residual structure. Secondly, the feature of different layers was reused to maximize the retention of small target insect feature by introducing Feature Multiplexing Module(FMM). Finally, Adjacent Elimination Module(AEM) was used to eliminate background information between adjacent feature layers that hinders the accuracy of small target pest recognition.

The main work of this paper was as follows:

1. The ability of convolutional kernel to extract detailed information about small target pests was tested. Compared with ResNet, S-ResNet model rarely used large convolutional kernels and replaced them with small convolutional kernels. Small convolutional kernels were more advantageous than larger in the extraction of small target insect detail features. Small convolutional kernels could retain richer detail features.

2. Benefiting from the inspiration of Single Shot MultiBox Detector(SSD), this paper analyzed the multi-level feature reuse module. The lower-level features retained more small target detail information, which was more beneficial to small target insect identification. In addition, the adjacent erasure module was also analyzed, where the feature layers gradually lose small target detail information during the forward propagation of the model. In order to retain the small target features, element-by-element subtraction was performed for the adjacent layers to suppress redundant information and improved the recognition accuracy of small targets.

3. In summary, an S-ResNet model was designed in this paper for identifying small target insects. To address the problems of insufficient feature extraction ability and excessive redundant background information of existing models for small target pests, the ResNet model was optimized to improve its feature extraction and retention ability for small target insects. Since S-ResNet was a model for classification tasks, it could be ported to detection or segmentation tasks as a backbone feature extraction model in the future.

## 2 Materials and methods

### 2.1 Image dataset

The size of small targets in this study was referred to the MS coco dataset (Lin et al., 2014), which considered that an image in which the actual target occupied pixels less than 32 × 32.

Images of 10 common insect species in the field were collected to construct the dataset, including Aphid, Red Spider, Locust, Sweet potato Whitefly, Rice Leaf Roller, Asian Rice Borer, Corn Borer, Land Tiger, Bollworm, and Cluster Caterpillar. Pest images were obtained from open access datasets such as Ip102 (Wu et al., 2019), Pest24 (Wang et al., 2020), or publicly available images on the internet. Images with insect targets pixel numbers more than that of the defined small targets were excluded. Finally, 250 images of each class of insects were included.

**Figure 2:**
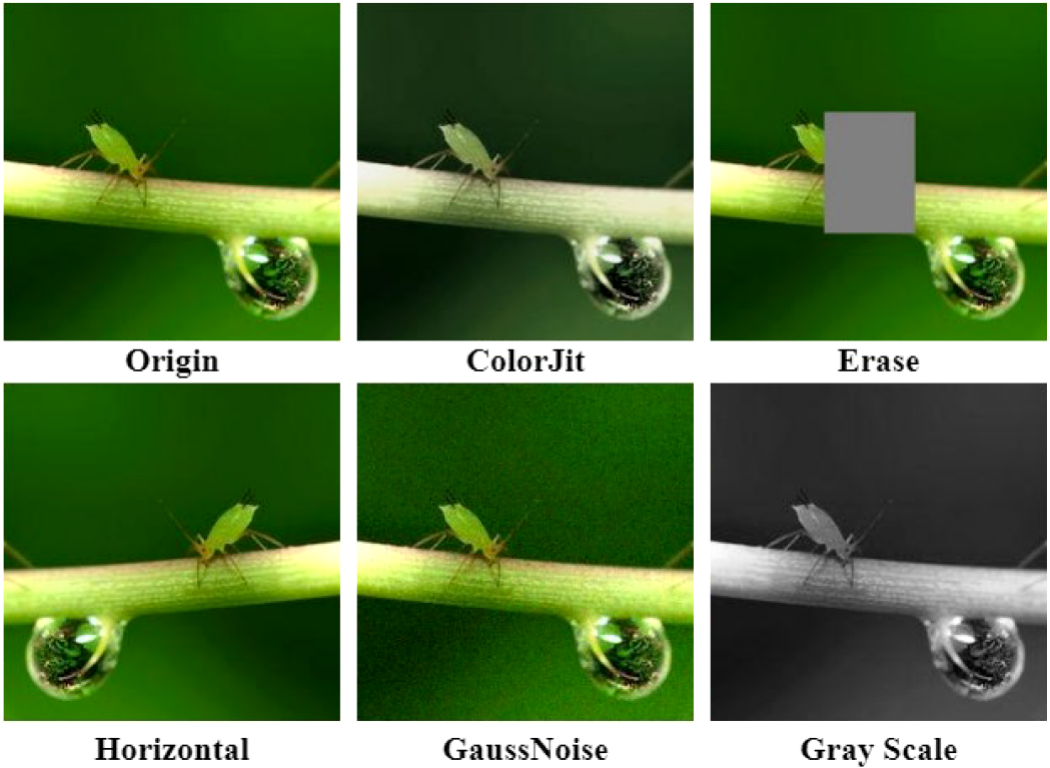
Data-augmentation operations of the original images.

CNNs performed well in many machine vision tasks. However, these models relied heavily on big data sources to avoid overfitting. Unfortunately, many application areas lacked big data sources, such as agriculture for small target insects. Therefore, data augmentation was one of the effective solutions to the problem of limited data sources. Data augmentation consists of a series of techniques used to increase the amount and quality of training datasets so that they could be used to build better deep learning models (Shorten and Khoshgoftaar, 2019). Krizhevsky et al. (2017) used data augmentation in their experiments to increase the size of the dataset by 2048 orders of magnitude. This was achieved by randomly cropping 224 × 224 blocks of regions from the original image, flipping them horizontally, and changing the intensity of the RGB channels using PCA color enhancement. This data enhancement helped reduce overfitting when training deep neural networks. The authors claimed that their method reduced the error rate of the model by more than 1%. To address the risk of underfitting due to insufficient amount of data sources, this study performed data augmentation operations on images of ten insect species. Each original image was expanded into 20 different images by common data augmentation means (e.g., rotation, random flip, brightness dithering, added noise, and **Figure 2** showed the results of data augmentation). Finally, 50,000 images were obtained. To balance the number of categories, each species of pest was given 5000 images, and the training and validation sets were divided in a 9:1 ratio (**Table 1**).

**Table 1:** Image amount of each insect species in the dataset (Random sampling from each category of insects).

Label	Class	Original	Expansion	datasets	
				Train(90%)	Validation(10%)
0	*Aphid*	250	5000	4500	500
1	*Red Spider*	250	5000	4500	500
2	*Locust*	250	5000	4500	500
3	*Sweet potato Whitefly*	250	5000	4500	500
4	*Rice Leaf Roller*	250	5000	4500	500
5	*Asian rice borer*	250	5000	4500	500
6	*Corn Borer*	250	5000	4500	500
7	*Land Tiger*	250	5000	4500	500
8	*Bollworm*	250	5000	4500	500
9	*Cluster Caterpillar*	250	5000	4500	500
Total	10	2500	50000	45000	5000

### 2.2 Convolutional neural networks

**Figure 3:**
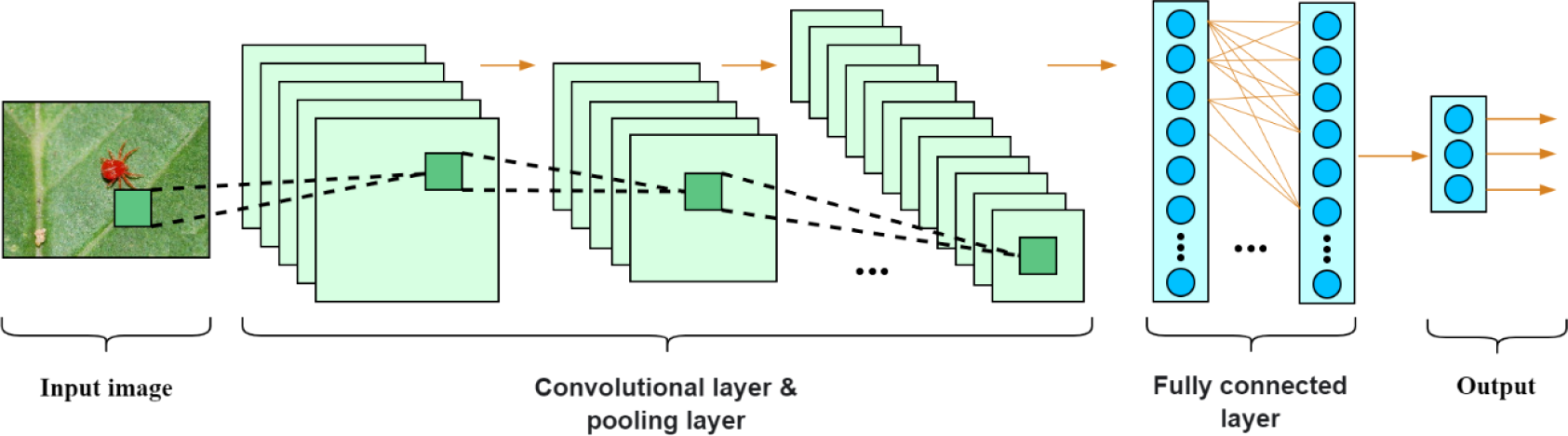
The structure of a common convolutional neural network.

The basic structure of a convolutional neural network was shown in **Figure 3**. It consists of several parts, such as input, convolutional layer, pooling layer, fully connected layer and output.

The mathematical principle of the convolution layer could be expressed as Equation 1. The convolution kernel (filter) slide through the layer’s feature layer in a specified stride and performed Hadamard multiplication with the corresponding feature map values according to a certain weight.


(1) $$y_{j}^{l}=f\left(\sum_{i\in F_{j}}x_{i}^{l-1}*k_{ij}^{l}+{\text{b}}_{i}^{l}\right)$$

where, *Fj* represented the convolution region of different feature maps; $b_{i}^{l}$ represented the bias term; *f* represented the added activation function that could add nonlinear factors to the network and thus improve the model’s expressiveness. The common activation functions for CNNs were ReLu, Sigmoid, etc.

The Pooling layer could effectively reduce the parameters transferred to the next layer of the model, improve the computational speed, and enhance the robustness to feature location shifts and deformations. The Pooling layer was commonly used with Maximum Pooling and Average Pooling. When the input size was *m×n* and the Convolution Kernel was *p×q*, Maximum Pooling formula could be applied as shown in Equation 2. When Average Pooling was applied, it would take the average of the specified region.


(2) $$\begin{array}{c} y_{ij}=\max(x_{i+r,j+s})\\ i\leq m-p\\ j\leq n-q \end{array}$$

### 2.3 ResNet network basic structure

**Figure 4:**
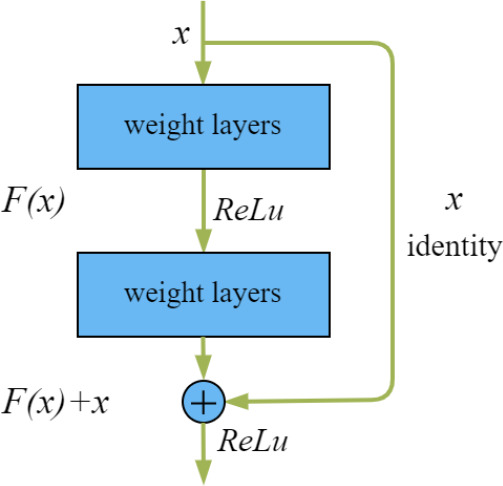
Residual structure.

The ResNet model had achieved excellent results on the ImageNet dataset (Deng et al., 2009; He et al., 2016). The residual structure (**Figure 4**) allowed the output from one layer of the network to be quickly transferred to the next or even more deeper layers using skip connections.

The principle of residual structure was shown in Equation 3. *H*(*x*) represented the output, and *F*(*x*) represented the result after the convolution layer. After introducing the residual structures, if *F*(*x*) became 0 during the forward propagation, the input *x* would automatically continue to pass through the branch of the identity so that the problem of degradation due to the deep network layers could be avoided.


(3) H(x)=F(x)+x

New models could be constructed by stacking residual structures into ResNet with different depths, such as ResNet-18, ResNet-34, and ResNet-50. The number of residual structure repetitions was shown in **Table 2**, where different of residual structures were stacked in Layer1 to Layer4. With a new convolutional layer added into each residual structure, ResNet50 would be generated from ResNet34, while they have the same times of stacked residual structure but different network depths.

**Table 2:** The amount of residual block repetitions for ResNet models of different depths.

Layers	Output	ResNet-18	ResNet-34	ResNet-50
Conv	112^2^	1	1	1
Layer1	56^2^	2	3	3
Layer2	28^2^	2	4	4
Layer3	14^2^	2	6	6
Layer4	7^2^	2	3	3
FC	10	1	1	1

FC means a Fully Connected Layer, which outputs the predicted probability in 10 classes of insects in one picture. The value in the table represents the amount of stacked residual modules in each model. For example, the ResNet-34 consists of 34 layers with one Conv, three residual structures in Layer1, four residual structures in Layer2, six residual structures in Layer3, three residual structures in Layer4 and one FC. Conv means a convolutional layer (containing BN layer and activation function).

## 3 S-ResNet

### 3.1 Network structure of the S-ResNet

**Figure 5:**
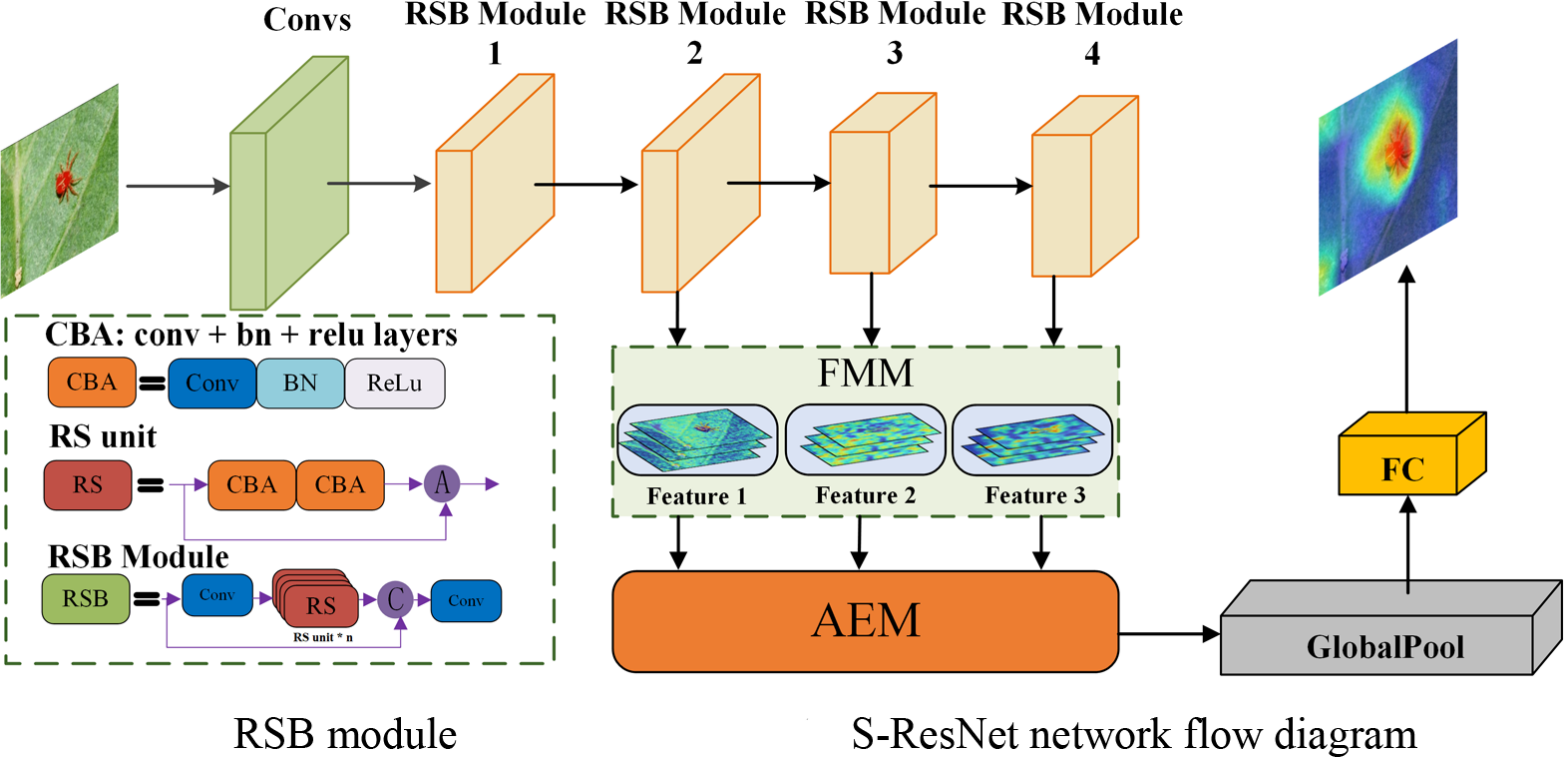
S-ResNet overall structure diagram. “A” donates the element-by-element summation; “C” donates the fusion of the number of feature channels; “Conv” donate a convolutional layer; “RS” donates a residual structure(The improved residual structure is shown in **Figure 7B**); “RSB” donates a RSB module; “BN” donates a normalization operation and “ReLu” is the activation function.

The conventional ResNet model did not have the high reuse rate for large-scale features. When identifying small insects, small-scale feature information could be easily lost during forward propagation, which would lead to false identification results (Liu et al., 2022). As there were few branches of the residual structures, the feature extraction and expression ability of the model should be limited (Qiu et al., 2021). Even more, there would be little extraction of deep semantic information. In this section, a feature extraction model called S-ResNet designed for small insect target identification. The model was improved from the ResNet model, by varying its convolution kernel. The branch of the residual structure was added, and the Feature Multiplexing Module (FMM) was illustrated. Therefore, the feature expression capacity of the model was improved using feature information of different scales. Meanwhile, the Adjacent Elimination Module (AEM) was furtherly employed to eliminate the useless information in the extracted features of the model. The general structure of the model was shown in **Figure 5**. The part of the model that consist of stacked residual blocks was defined as Residual Body Module (RSB). The amount of stacked residual structures in the RSB module varies in different ResNet model as presented in **Table 2**. The improved residual structure was used for feature extraction of small insect targets. At the same time, to prevent feature loss of small target pest information during propagation. A branch was added before each RSB module, which was fused with the output content of RSB after convolution operation to enhance the feature extraction ability of small insect targets. Then the AEM operation was performed by sequentially outputting features of different dimensions, furtherly extracting the feature information of small insect targets. Finally, the feature channel depth was reduced *via* the convolutional layer. The probability of the output category was achieved after Global Average Pooling, Fully Connected Layer, which would indicate the classification results of each insect species.

### 3.2 Reduction of the convolution kernel size

The ResNet model used a 7×7 convolutional kernel as the steam for feature extraction. It was easy to recognize large targets in the image because of its larger perceptual field and original image mapping area. However, detailed information of the small insect targets would be lost during convolution procedures with the 7×7 convolutional the kernel, making the identification of small insect targets more difficult. Multiple small convolutional kernels were applied in this study instead of large convolutional kernels according to the algorithm proposed by Simonyan and Zisserman (2014). Using ResNet-34 as the benchmark, ablation experiments were conducted to select proper number of 3×3 convolutional kernels for small insect target identification. The test results were shown in **Table 3**.

**Table 3:** The effect of convolution kernel number on the ResNet-34 model.

Trial	Number of 3×3 convolutions	Number of 7×7 convolutions	Accuracy/%	Model Size/MB
1	0	1	88.4	87.33
2	1	0	88.6	**86.87**
3	2	0	**88.7**	87.06
4	3	0	**88.7**	87.21

Note: Bolded font represented the best result in a column.

As presented in **Table 3**, the accuracy of the ResNet-34 model was improved after using the 3×3 convolution kernel, where using three 3×3 convolution kernels could achieve the same perceptual field as a 7×7 convolution kernel. The recognition accuracy was increased from 88.4% to 88.7%. Meanwhile, the model size was slightly reduced.

**Figure 6:**
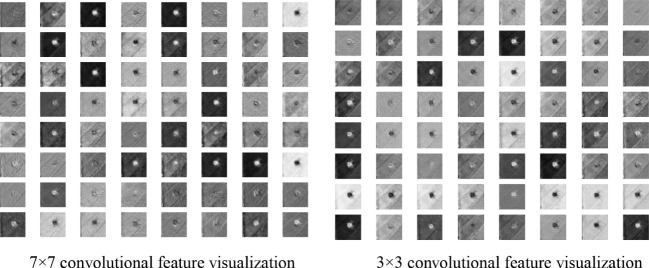
Feature visualization using a 7×7 and a 3×3 convolution kernels.

The visualization operation of the feature obtained from the 7×7 and 3×3 convolution kernels were shown in **Figure 6**. It showed that the 3×3 convolution kernel could get a relatively clear outline of the small insect target. Therefore, a 3×3 convolution kernel was used to replace the 7×7 convolution kernel in the first layer of the original model. The improved model was less likely to lose the detailed information of small insect targets due to the smaller perceptual field.

### 3.3 Residual structure improvement

**Figure 7:**
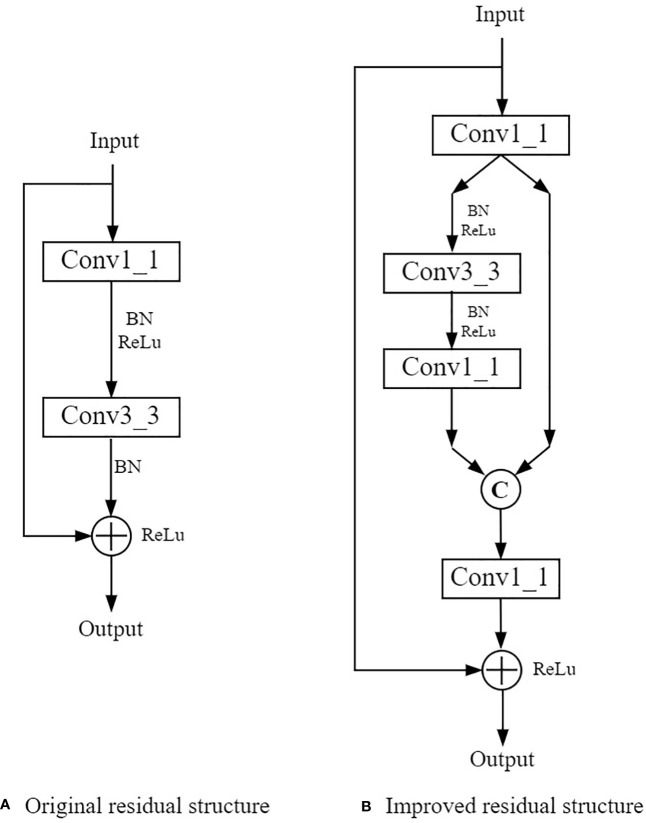
The original residual structure **(A)** in ResNet model and the improved residual structure **(B)** in S-ResNet model. Conv3_3 and Conv1_1 donate convolution operations with the convolution kernel size of 3×3 and 1×1 respectively; BN donates a normalization operation; ReLu is the activation function;“C” donates the fusion of the number of feature channels; "+" represents the summation of feature maps.

Addition of branches to the residual structure could improve the feature extraction ability of a model generated from ResNet (Ren et al., 2019; Bao et al., 2021). The residual structure was improved in this study to strength the feature extraction ability of the model for small insect targets, as shown in **Figure 7**. The improved residual structure contained three branches. The input feature underwent a dimensional boost operation with a 1×1 convolution kernel, in which the resolution of the feature did not change and the depth of channels was doubled. Then the feature was divided equally into two branches as shown in **Figure 7B**. The left branch underwent further feature extraction with 3×3 convolution and 1×1 convolution operations, while the right branch did the skip connection operations. Output of the two side branches were formed into a new feature using channel fusion operation. Then, the dimensionality of its fused feature was reduced with a 1×1 convolution kernel. The output was then added with the feature of the branch directly from Input for a fusion with the element-by-element summation method as shown in **Figure 7B**. With this kind of operation, the feature information of the small target was retained in maximal extent. The improved residual structure could extract and express feature information at different scales more effectively than the original model, while avoiding the problem of small insect target information loss in the forward propagation.

### 3.4 Feature multiplexing module and adjacent elimination module

**Figure 8:**
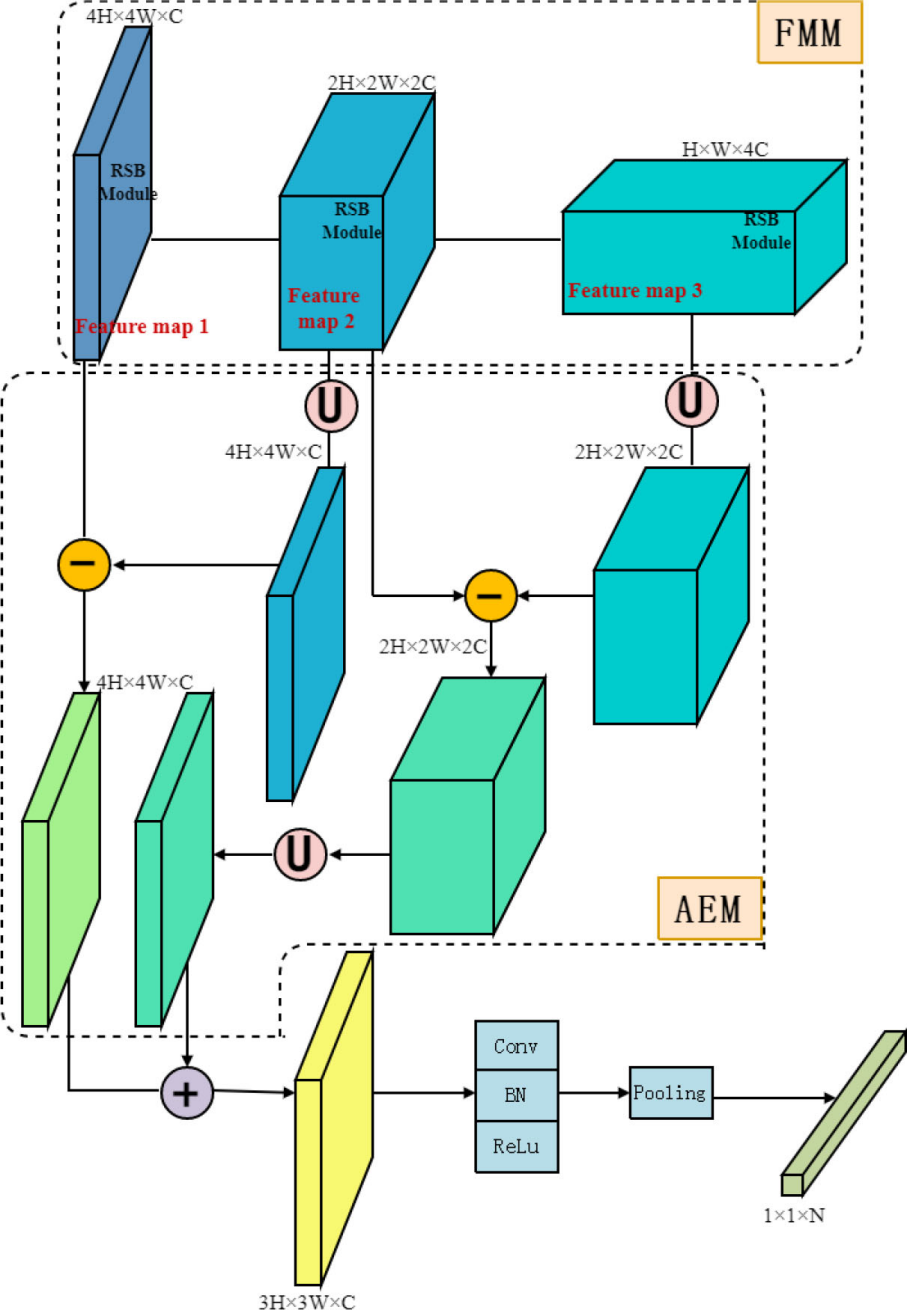
Feature Multiplexing Module and Adjacent Erasing Module. “U” represented an upsampling operation to expand the resolution of the feature map by a factor of two and a 1×1 convolution operation to reduce the number of channels; “-” represented an element-by-element subtraction operation for the feature map; “+” represented an element-by-element addition operation for the feature map.


The deep learning model could extract different feature information at different convolutional layers during forwarding propagation, which could be used to identify various target sizes. The target detail information decreased while the affluent semantic information increased as the model move from shallow to deep layers. Liu et al. (2016) proposed an SSD model in which the feature extracted from the backbone was multiplexed to detect targets in various sizes. The Feature Multiplexing Module (FMM, **Figure 8**) based on the above idea was introduced in the model of this study to improve the feature reuse. The features extracted by the RSB2 to RSB4 modules were reused when they were input into the Adjacent Elimination Module (AEM, **Figure 8**) recording to Neighbor Erasing and Transferring Network (NETNet) proposed by (Li et al., 2020), which has achieved good results in detecting small targets on the MScoco dataset. The AEM was an operation between two adjacent network layers. When the size of a feature image in layer m was described as h*_m_* ×w*_m_* ×c*_m_*, the feature information in layer *l* should be *f*_*l*_={*x*_*l*_,*x*_*l*+1_,*x*_*l*+2_,…,*x*_*n*_}∈*R* ^*h*_*l*_×*w*_*l*_×*c*_*l*_^ , and the feature information in layer *l+1* should be *f*_*l*+1_={*x*_*l*+1_,*x*_*l*+2_,*x*_*l*+3_,…,*x*_*n*_}∈*R* ^*h*_*l*+1_×*w*_*l*+1_×*c*_*l*+1_^ , where *h_l_ > h_l+_*_1_ and *w_l_ > w_l+1_*. Usually, during the forward propagation from *f_l_* to *f_l+1_*, the reduced part *x_l_* might contain the detailed information of the target. Due to little detailed information on small insect targets, they could be easily overlooked by the model during forward propagation. Therefore, the element-by-element subtraction of the feature at different scales using Equation 4 to eliminate redundant background features and retain target information.

(4) $$f_{s}^{\prime }=f_{l}⊖f_{l+1}=\left\{x_{l},x_{l+1},x_{l+2},\ldots ,x_{L}\right\}⊖\left\{x_{l+1},x_{l+2},x_{l+3},\ldots ,x_{L}\right\}$$

Specifically, the output of the RSB4 was subjected to an Upsampling operation to double its feature size to ensure that the feature could be subtracted element by element. Then the depth of the feature was reduced using a 1×1 convolution filter. The output of the RSB3 was subjected to feature erasure to extract information of small insect targets. Similarly, the output of RSB3 was feature-erased with the output of the RSB2 module after Upsampling operation. Then the results obtained from the two feature erasures were summed element by element to get the final output feature. After a series operation of convolution, Pooling, and activation function, Fully Connected Layers were employed for classification.

## 4 Experiments

### 4.1 Setup

The hardware platform used for the experiments was a desktop computer with a NVIDIA RTX 2060 GPU unit. The operating system was Linux Ubuntu 20.04. The CUDA version was 10.1. The source code of the neural network was implemented in Python under the framework Pytorch. The initial learning rate was 0.0001. The training model epoch was set to 200. The model was optimized using the Adaptive Moment Estimation (Adam) optimizer. The batch size was 64 for training and 32 for validation in each epoch.

The model performance was evaluated with accuracy, precision and recall. To furtherly measure the model’s merit, the model size was also used to assess the complexity of the model. Cross-Entropy Loss Function which was closely related to the probability distribution of events was considered as the loss function as in Equation 5.


(5) $$L=\frac{1}{N}\sum_{i}L_{i}=-\frac{1}{N}\sum_{i}\sum_{c=1}^{M}y_{ic}\log(p_{ic})$$

### 4.2 Impact of augmented dataset on model performance

**Figure 9:**
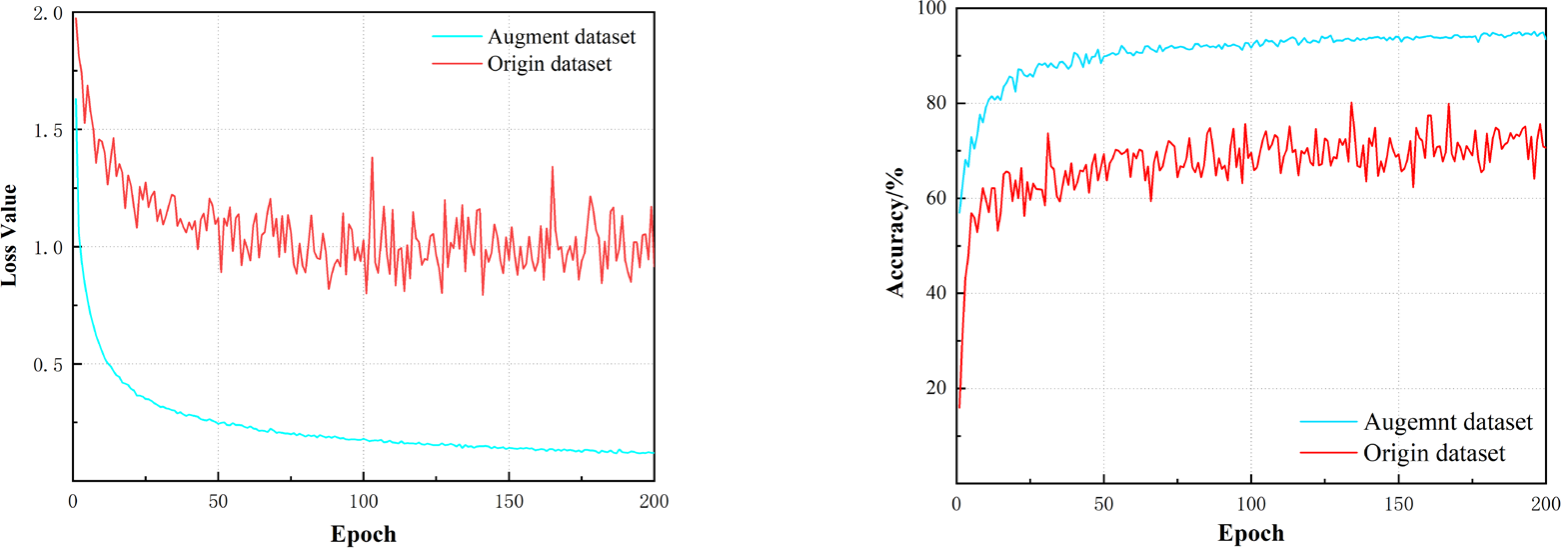
Loss Value and Accuracy changes of S-ResNet34 on the datasets before and after expansion.

To verify the effect of data augmentation on model training, S-ResNet 34 was employed for model training respectively with the original dataset and the augmented data in this study. The experimental parameters were set as described in Section 4.1. The results of the loss values on the training dataset and the accuracy variation on the validation dataset were shown in **Figure 9**. The maximum classification accuracy of the model trained with original dataset was 81.47%, while that of the model trained with augmented dataset was 95.26%. Meanwhile, the training procedure with augmented dataset converged faster and processed more smoothy. Therefore, the following experiments in this study were conducted with the augmented dataset.

### 4.3 Effect of improved residual structure on model performance

Several types of S-ResNet and ResNet models with different layers were compared and tested without adding FMM and AEM to verify the impact of the improved residual structure on the model identification performance. The comparison results were listed in **Table 4**.

**Table 4:** Impact of improved residual structure on network performance.

Trial	Model name	Number of improved residual structures	Accuracy/%	Precision/%	Recall/%	Model Size/MB
1	ResNet18	0	86.7	86.7	85.9	46.8
	S-ResNet18	8	**89.6**	91.2	90.1	43.5
2	ResNet34	0	88.4	88.7	86.3	87.3
	S-ResNet34	16	**90.3**	91.4	89.4	82.6
3	ResNet50	0	90.6	91.3	89.3	102.6
	S-ResNet50	16	**90.4**	92.9	90.5	98.6
4	ResNet101	0	89.9	90.7	87.3	169.9
	S-ResNet101	33	**90.8**	91.8	91.3	160.6

Note: The bold font represented that the S-ResNet model which added the improved residual structure had higher accuracy compared to the original ResNet model.

**Figure 10:**
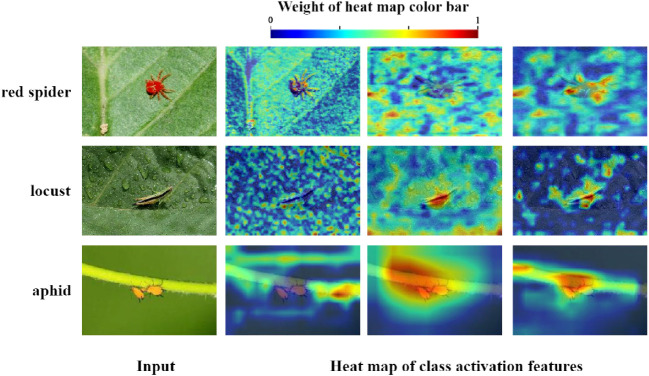
Heat map visualization with S-ResNet34 feature extraction module. The heat maps of class activation features in b are the output of the 2nd, 3rd, and 4th RSB modules (from left to right). Red pixels represent the area of concern of the model while the blue pixels represent the area of considered background.

When the improved residual structure was added to the ResNet model, the performance of all aspects was better than the original ones, in which the accuracy of the 18-layer ResNet network increased with greatest variation of 2.9%. However, with the increase of model depth, the model recognition accuracy did not rise significantly. The recognition accuracy even decreased in ResNet101 model. To furtherly demonstrate the feature extraction capability for small insect targets, this study used Gradient-weighted Class Activation Mapping(Grad-Cam) (Saini et al., 2020) for visualization operations, which showed the essential regions of the image used for model prediction. The outputs of the second, third, and forth RSB modules were visualized using Grad-Cam as shown in **Figure 10**. With the increase of forwarding propagation depth, the model did not entirely focus on the target location in the images. Instead, a large amount of background information was mixed, which resulting in the insignificant increase in the accuracy of small insect target identification. Therefore, model optimization to enhance the small target insect feature information extraction was necessary.

### 4.4 Impact of FMM modules and AEM on network performance

**Figure 11:**
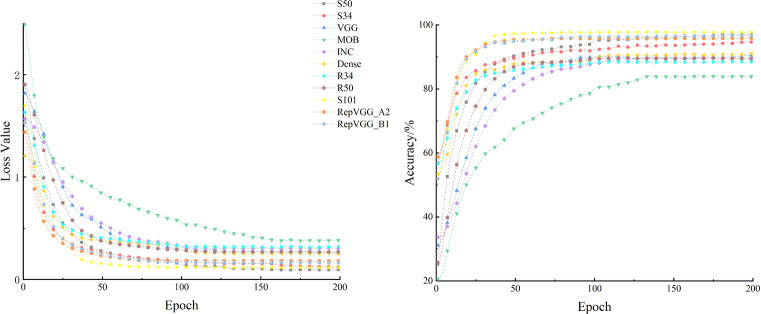
Variation of loss values and accuracy of different models on the validation set(Smoothing value=0.6).

Different feature information from the RSB module was processed in the FMM and AEM modules, after which the network suppressed irrelevant background information and would focus on small target pest feature information. The modules were added to the ResNet model (i.e., the S-ResNet model designed in this study) and compared with the model before improvement on their feasibility of the feature processing. The test results were presented in **Figure 11** and **Table 5**.

**Table 5:** Impact of feature processing modules on model performance.

Trail	Model name	FMM+AEM	Accuracy/%	Model Size/MB
1	ResNet18	–	86.7	46.8
	S-ResNet18	√	93.6	49.5
2	ResNet34	–	88.4	87.3
	S-ResNet34	√	95.3	91.2
3	ResNet50	–	90.6	102.6
	S-ResNet50	√	**97.2**	105.5
4	ResNet101	–	90.8	169.9
	S-ResNet101	√	**97.8**	173.2
5	VGG-16	–	90.5	218.8
6	Inception_v3	–	91.2	92.9
7	MobileNet_v3	–	84.3	16.1
8	DenseNet121	–	92.3	30.8
9	RepVGG_A2	–	95.8	112.8
10	RepVGG_B1	–	96.6	229.7

Note: The bold font represented the higher accuracy of the model proposed in this paper compared to other models.

**Figure 12:**
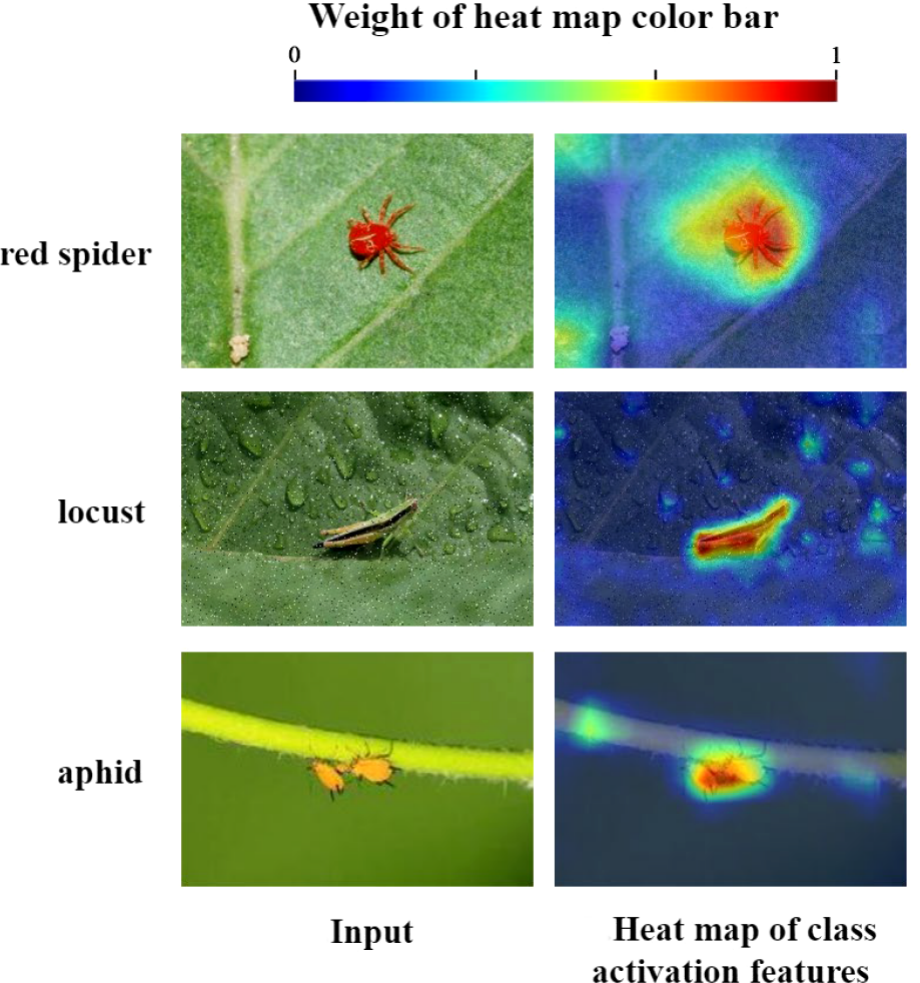
Heat map of the output after adding the feature processing module.

The accuracy of each model stabilized on the validation set after 200 epochs (**Figure 11**), which indicated that the performance of the model had been fully demonstrated. The MobileNet had the worst performance in these models. The S-ResNet101 and RepVGG model showed similar convergence speed, but S-ResNet101 was higher accurate. The S-ResNet101 and S-ResNet50 model were basically the same in terms of accuracy. There are two possible reasons. 1) It was possible that the current amount of data was not sufficient for the large size of the model. 2) Even though the improved model had significant advantages for small target insect feature extraction, there were still some problems that need to be explored in the future. In a word, it could be seen that the recognition accuracy of the model on the validation dataset was significantly improved after adding the feature processing module. And the S-ResNet model in this study had better convergence speed than other models. The output of the S-ResNet34 model was visualized using Grad-Cam (**Figure 12**). The final output of the model focused on the target pest area rather than the background area. Thus, the introduction of FMM and AEM modules could be practical for accuracy improvement of the model on identifying small insect targets.

### 4.5 Identification results of different categories of insects

**Figure 13:**
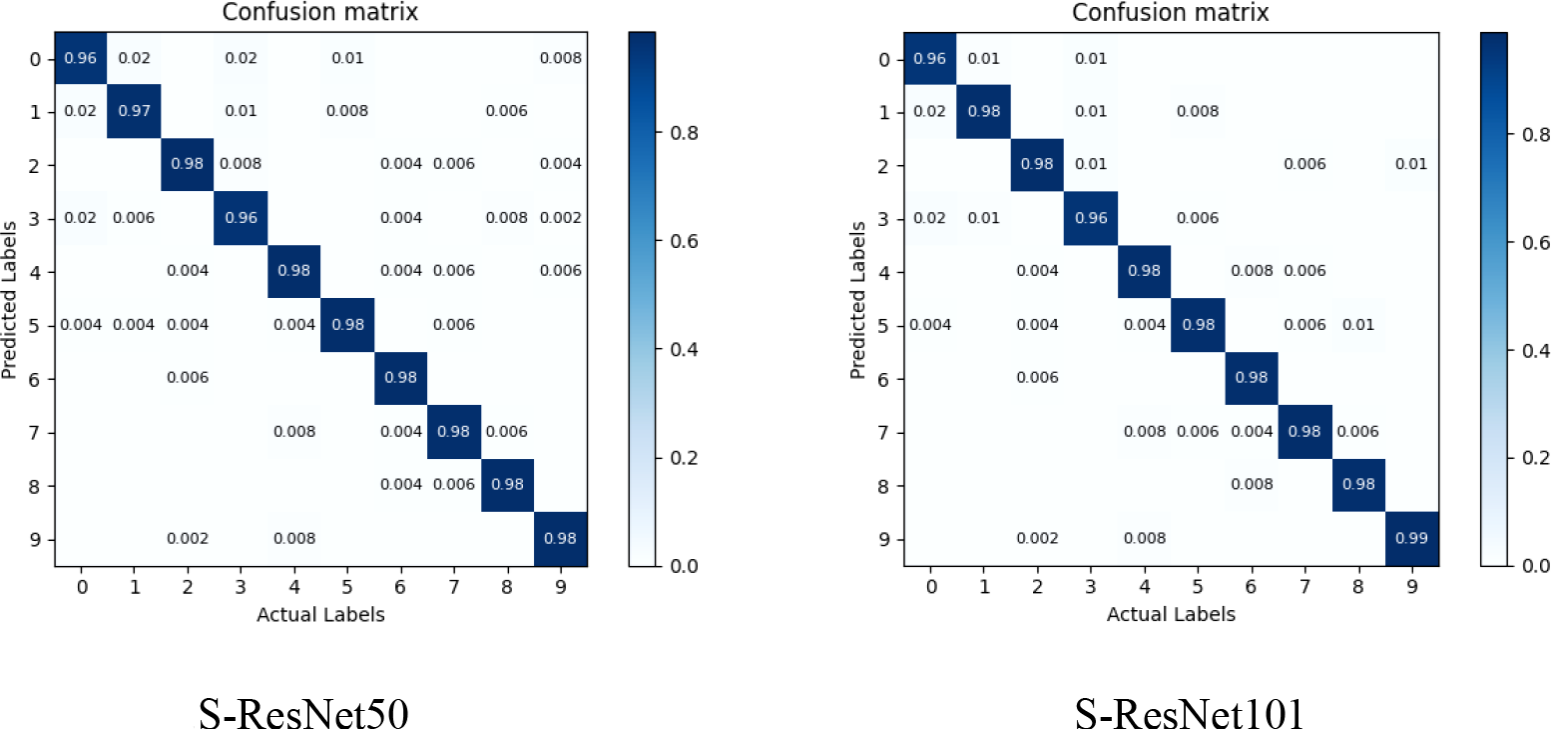
Confusion matrix for the identification results of 10 classes of small insect targets. Aphid (0), Red Spider (1), Locust (2), Sweet potato Whitefly (3), Rice Leaf Roller (4), Asian Rice Borer (5), Corn Borer (6), Land Tiger (7), Bollworm (8), and Cluster Caterpillar (9).

The S-ResNet50 and the S-ResNet101 model was tested on the validation dataset in this study to analyze the improved model’s effectiveness on recognizing small insect targets. The validation results were shown in a confusion matrix (**Figure 13**). Labels 0 to 9 correspond to 10 different insect species, specifically: Aphid (0), Red Spider (1), Locust (2), Sweet potato Whitefly (3), Rice Leaf Roller (4), Asian Rice Borer (5), Corn Borer (6), Land Tiger (7), Bollworm (8), and Cluster Caterpillar (9).

The results showed that the model achieved good recognition accuracy for most insect species, while labels 0, 1, and 3 had slightly lower recognition accuracy compared to other species (Label 1 performed better on S-ResNet101 model than S-ResNet50). This could be attribute to their living environment where these pest targets were relatively small or similar to the natural background color.

## 5 Discussion

Crop pests were one of the main risk of yield loss to agriculture production. Automatic, fast and accurate identification of pests was essential for infestation level prediction of pests in the field and the improvement of integrated pest management strategy. In this study, we proposed a recognition model for small target insects in the field. Compared with the ResNet model and other advanced models, the model in this paper was able to achieve better recognition accuracy with low additional overhead on small target insect datasets.

As can be seen in the confusion matrix (**Figure 12**), the category of labels 0 and 3 still suffers from a lack of recognition accuracy (accuracy of about 96%) with insects of small size and similarity to their environment. There were many reasons for this, as the dataset size could be one of the major impact factors. Compared to ImageNet and COCO datasets which had millions of images, the existing small target insect dataset had less image data. Expanding of image data frame could benefit to fitting the large model training requirements and achieve the desired insect identification results. In the DPeNet model-based automatic insect monitoring system developed by Zhao et al. (2022), 325 original images were collected and expanded to 22,815 for model training by means of data enhancement. Compared with Muscidae (99.1%), Araneae (100%), Apis (100%) and other insects with larger target sizes (1.5-2 cm), the trained model in this study had the recognition rate of 97.7% for small target pests (1-3 mm). In the dataset of Pest24 (Wang et al., 2020), the recognition of insects with small size and little images was also poor for all types of detection models (AP below 30%). In contrast, the AP could reach more than 90% for some insects in large size and with rich images in the dataset. Meanwhile, insects had different degrees of damage to crops in different life cycles. To achieve insect prevention, images of insect classes at various growth stages need to be collected. In the dataset of IP101 (Wu et al., 2019), images of insects at different growth stages including eggs, larvae, pupae and adults were collected. However, it was also a serious challenge for model recognition due to different appearance features. In summary, the size and number of insects had significant impact on the recognition effect of the model. To ensure a uniform data distribution, each small target pest category in this study had the same number of images. However, in the presence of a large number of pest species in agriculture, ten categories of pests were still difficult to meet the requirements of crop prevention and monitoring pests. A richer and more diverse dataset of small target insects was one of the important means to ensure accurate crop prevention and control.

In the natural environment, it was difficult for the model to locate the main position of the insect due to the interference of complex background (**Figure 10**). Liang et al. (2022) collected rice insect images in different scenarios (field environment, trap light captures and indoor whiteboard) and used Yolov5 to detect insects in these three scenarios. The results showed that the model had a lower recognition rate for insects acquired in the field and trap light scenarios and a higher recognition rate for indoor whiteboard insects. This result was related to the training background where the rice background taken in the indoor whiteboard background was single, less distracting and with clearer pest features. In contrast, field backgrounds were diverse, more complex and could be affected by various factors such as light, slope and cultivation. Qiu et al. (2021) found that recognition rates were affected by the sampling background in grassland coveted night moth recognition counts (pest recognition accuracy was higher on a white background than on a circular grid background). The FMM+AEM module introduced in this study effectively eliminated the background information (**Figure 11**) and enhanced the recognition accuracy of small target pests. However, the dataset in this paper contained fewer insect targets in a single image. Thus, further testing of the model performance was needed when facing issues such as dense insect population and high adhesion level. Meanwhile, the simple classification task could hardly meet the demand for multi-class insect prediction and monitoring. The more advanced detection task performed better in pest warning. As the high modularity of deep learning models, it was cheaper to pre-train the backbone feature extraction model and then port it to detection or segmentation tasks.

Timely and accurate identification of insects was an important prerequisite for effective control. Existing convolutional neural network-based pest identification models suffer from poor real-time performance and complex structures could not be easily deployed. Although the Pest-YOLO (Wen et al., 2022) was effective for detecting dense and tiny pests, the model was run in a specific environment. Limited by the performance of hardware, it still remains a difficult problem to enhance the model maintain recognition performance while minimizing its number of parameters. Lightweight models have attracted more researchers’ attention because of their great advantages in inference speed and number of parameters. Peng et al. (2022) proposed a SNPF lightweight model based on the ShuffleNet V2 model. By adjusting the number of output channels of the original model and the number of core module stacks, the SNPF model identified agricultural insects with higher accuracy (4% improvement), faster (11.9 ms inference time) and lower number of parameters (30.6% reduction). The lightweight design of the model is also one of the next priorities of this research direction, so that it can meet the deployment requirement on portable mobile devices.

## 6 Conclusion

In this study, an S-ResNet model was proposed for identifying small crop insect targets. The proposed method contains three key components: the optimization of the residual structure, the modification of the convolutional kernel and the introduction of the FMM+AEM module. The S-ResNet model proposed in this paper showed a significant improvement in the accuracy of recognizing small target pests compared to the ResNet model (the highest S-ResNet101 achieves 97.8% recognition accuracy, with improvement of 7%) through comparison tests with 10 types of pest image collection. Compared with other advanced deep learning models, the model in this study maintains its advantage in recognizing small target pests. The model proposed in this study could provide an alternative technique for monitoring and precision control of small target insect in future.

## Data availability statement

The datasets in this article are collected from publicly available datasets. This data can be found here: https://github.com/xpwu95/IP102.

## Author contributions

HL, FL, and PW contributed to conception and design of the study. HL and FL organized the database. HL, FL, LW, and QN performed deep learning and the statistical analysis. FL wrote the first draft of the manuscript. HL, QN, CL, and PW wrote sections of the manuscript. All authors contributed to the article and approved the submitted version.
